# Colony-level aggression escalates with the value of food resources

**DOI:** 10.1186/s12862-023-02117-x

**Published:** 2023-05-16

**Authors:** Shaolin Han, Ben L. Phillips, Mark A. Elgar

**Affiliations:** 1 School of Biosciences, University of Melbourne, Melbourne, VIC 3010 Australia; 2 Present Address: School of Biological Sciences, University of Hong Kong, Hong Kong SAR, China; 3 Present Address: Centre for Immunology & Infection, New Territories, Hong Kong SAR, China

**Keywords:** Aggression, Escalation, Group contest, Social insect

## Abstract

**Background:**

Theory predicts that the level of escalation in animal contests is associated with the value of the contested resource. This fundamental prediction has been empirically confirmed by studies of dyadic contests but has not been tested experimentally in the collective context of group-living animals. Here, we used the Australian meat ant *Iridomyrmex purpureus* as a model and employed a novel field experimental manipulation of the value of food that removes the potentially confounding effects of nutritional status of the competing individual workers. We draw on insights from the Geometric Framework for nutrition to investigate whether group contests between neighbouring colonies escalate according to the value to the colony of a contested food resource.

**Results:**

First, we show that colonies of *I. purpureus* value protein according to their past nutritional intake, deploying more foragers to collect protein if their previous diet had been supplemented with carbohydrate rather than with protein. Using this insight, we show that colonies contesting more highly valued food escalated the contest, by deploying more workers and engaging in lethal ‘grappling’ behaviour.

**Conclusion:**

Our data confirm that a key prediction of contest theory, initially intended for dyadic contests, is similarly applicable to group contests. Specifically, we demonstrate, through a novel experimental procedure, that the contest behaviour of individual workers reflects the nutritional requirements of the colony, rather than that of individual workers.

**Supplementary Information:**

The online version contains supplementary material available at 10.1186/s12862-023-02117-x.

## Background

Conventional aspects of animal contests have been explained by game theory, initially predicting that selection favours signal displays rather than fatal and physical fights, and thus contests are typically resolved without injuries [1, 2]. However, theory also predicts that individuals may accommodate the costs associated with escalation according to the value of the contested resource [1, 3–9], and empirical support is provided by studies of dyadic contests, especially where males compete for mating opportunities [10–14], females compete over oviposition sites [15–17] and nests [18–20] and, less extensively, individuals compete over food [21]. Despite this wealth of empirical work there have been few tests of this theoretical prediction for contests involving groups of individuals. While aggression associated with resource value in group contests has been documented in some species, including primates [22] and social insects [23, 24], the effect of resource value on levels of escalation in group contests has not been investigated experimentally. Manipulating the value of food resources in group contests is challenging because, first, the value to each contestant can differ, depending on the intrinsic features of the food [25] and, more importantly, contestants may differ in physiological state and prior experience [8, 21]. For example, the value of food may be greater to a food deprived than satiated contestant [9], irrespective of the quality or quantity of the food items. Another challenge is that, in a group, it is unlikely that all members of the group share the same foraging history and obtain the same nutritional state, so these individual differences may obscure any group-level patterns.

However, the Geometric Framework [26] provides a useful tool for experimentally manipulating the value of food resources in animal contests. A key prediction of the Geometric Framework is that individuals meet nutritional targets by compensatory foraging, where their current, preferred macro-nutrient intake depends upon their prior acquisition. Taxonomically broad empirical support for compensatory foraging is provided primarily by short-term experiments with captive individuals of both solitary [27–31] and social species, including honey bees [32] and ants [33–38]. Free-living ant colonies, with continual access to their typical diet, nonetheless vary their deployment of workers to foraging sites, based on their foraging history [39–41], and such homeostasis is thought to be maintained at the colony level [42]. For example, colonies of *Iridomyrmex suchieri* deployed more workers to an artificial protein source if they had been previously fed carbohydrates rather than protein [40], suggesting that the value of protein to the colony depends on its prior dietary history. Accordingly, it is possible to design staged contest experiments in which the same food is provided across all treatments, but the value of the contested food can be altered by manipulating the prior dietary history of the contestants.

Here, we address the applicability of contest theory for group contests by investigating whether levels of escalation in a social insect, the meat ant *Iridomyrmex purpureus*, are influenced by the value of a contested food resource, as manipulated by colony dietary history. Neighbouring colonies of *I. purpureus* deploy numerous workers to display grounds, located between their nests, where pairs of non-nestmates engage in typically non-injurious agonistic displays [43]. While lethal fights are rare at these display grounds, escalated interactions may still occur [43, 44]. The display grounds do not mark territorial boundaries, as previously inferred [45–48], but rather reflect contests over resources, including food trees [49] that support honeydew-secreting hemipterans, which are harvested by workers of *I. purpureus* and form a significant component of their diet [46, 47, 50, 51]. The level of aggression between displaying workers contesting access to food trees is higher closer to the base of the food tree than that at the display ground that is typically located midway between the neighbouring nests [49], suggesting that contest escalation may be linked to food resources.

First, we test whether field colonies of *I. purpureus* are sensitive to their past intake of carbohydrate and protein and compensate their current intake accordingly by conducting compensatory foraging assays. Second, we use these insights to manipulate, for the staged contests between neighbouring colonies, an appropriate contested food resource and dietary history. This novel, two-stage field experimental design allowed us to ask whether the deployment and behaviour of workers contesting a food resource are influenced by its nutritional value to the colony, as predicted by conventional contest theory.

## Methods

**Fig. 1:**
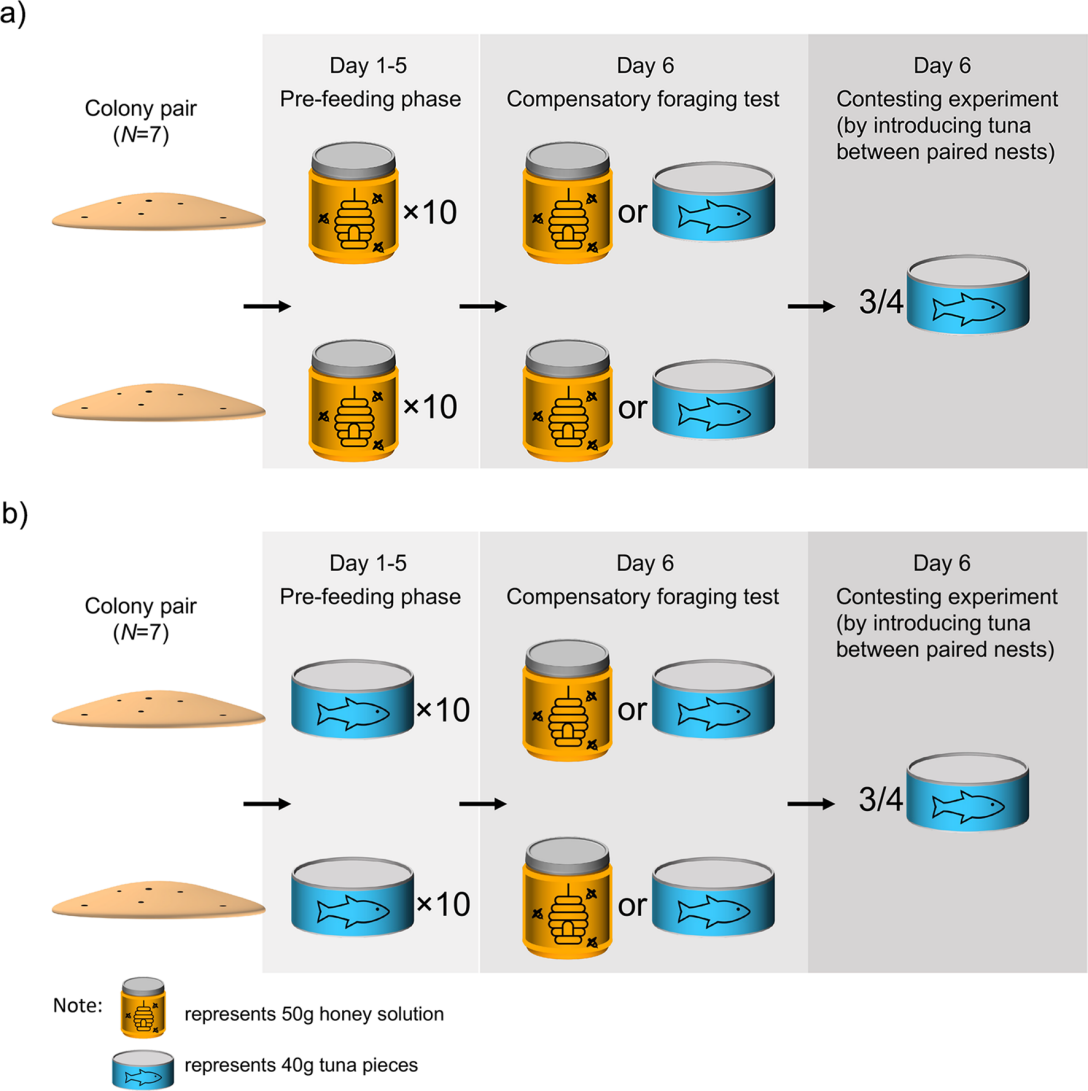
Experimental design of staged contests. Colony pairs assigned to **a** high value food or **b** low value food were fed honey solution and tuna, respectively, for five days during the pre-feeding phase. On day six, each colony within the pair was fed either honey solution or tuna to confirm a compensatory foraging effect. A contest over food was then initiated (about 1 h later) between the colony pair by placing tuna onto the active display ground between the pair of colonies

Initially, we investigated how field populations of meat ants *I. purpureus* adjusted their foraging effort in order to determine the experimental protocol for the staged contests. We tested, in early summer (November) 2019, for compensatory foraging between carbohydrate and protein, using a combination of a pre-feeding phase and a testing phase. Then we conducted staged contest experiments over two summers from 2020 to 2021 (early summer: February 2020, November 2021, February 2021; late summer: May 2020, May 2021). These field experiments involved three stages: a pre-feeding regime (to establish the ‘value’ of the contested food), a compensatory foraging test (to confirm consistent patterns of compensatory foraging) and a staged contest (to measure the level of escalation according to the value of the food) (see Fig. 1). All experiments were undertaken on rainless days, thus eliminating the impacts of rain on ant activities.

### Study site

Field experiments were conducted at Serendip Sanctuary (38° 00′ 03"S, 144° 24′ 42"E), located approximately 60 km southwest of Melbourne. The 250 ha of reclaimed farmland consists of semi-disturbed open woodland and grassland and supports many hundreds of colonies of meat ants *I. purpureus*. This widely distributed species is documented as polydomous [52], but this colony structure is rare at Serendip Sanctuary, where the large population of colonies is densely distributed [43].

### Pre-feeding phase

We selected 14 neighbouring colony pairs (28 colonies in total) that shared an active display ground and randomly assigned each pair to either a carbohydrate or protein pre-feeding treatment group (7 colony pairs for each group). For the first five days (pre-feeding phase), the carbohydrate group was provided with uncontested access to a feeder containing carbohydrate and the protein group was provided with uncontested access to a feeder containing protein. Each feeder was placed adjacent to the nest mound of each colony at 9.30am, replenished at 1.30 pm and removed at 5.30 pm. The carbohydrate source was 50 g of 25% honey (Woolworths™ branded ‘pure honey’) solution soaked in a cotton pad (9 cm diameter) and placed on a paper plate (15 cm diameter). The protein source was 40 g of small morsels of tuna (Woolworths™ branded ‘canned tuna in springwater’) shaped into a 9 cm diameter disk and placed on a paper plate (15 cm diameter). The stated nutritional content (per 50 g) of the honey solution is 10.39 g carbohydrate; less than 0.13 g protein; less than 0.13 g fat; 0.0019 g sodium, and the stated nutritional content (per 40 g) of tuna is less than 0.04 g carbohydrate; 10.04 g protein; 0.4 g fat; and 0.08 g sodium. Thus, the diets differ in the relative quantity of carbohydrate and protein. As these food items also differ in sodium and fat, it is possible the colonies are responding to a combination of these four macronutrients. However, the amount of sodium and fat is relatively minute and thus seems unlikely to contribute [40]. Additionally, as our study site is 5 kms away from the coast, sodium is unlikely to be limiting in our system [53].

### Compensatory foraging test

On the 6th day (the testing phase), colonies within each group were randomly divided into two subgroups (7 colonies per subgroup), with one subgroup provided with uncontested access to honey solution and the other to tuna pieces. The feeder (same as above) was placed (using surgical gloves to minimize any disturbance effects of human odour) adjacent to each colony nest mound at 9.30am, and we obtained digital photographs of each feeder at 10.00am, 10.30am, 11.00am, and 11.30am, after which the feeder was removed. The number of ant workers on each image was counted, blind to the time interval and treatment.

### Analysis of compensatory foraging test

Data collected in November 2019 were analysed to determine the experimental protocol for the staged contests. We used Linear Models to investigate the influences of pre-feeding phase and testing phase on the (log transformed) number of workers at the feeder. There was a pre-feeding treatment by testing treatment interaction term (F_1, 24_ = 8.40, *p* = 0.008; see Additional file 1: Fig. S1 for November 2019), indicating a clear effect of pre-feeding in response to the testing food, so the data were analysed separately for each testing treatment. These separate models revealed that the pre-feeding treatment influenced the number of workers deployed to the tuna in the testing phase, with significantly higher numbers of workers from colonies that had been pre-fed with honey solution than with tuna (Wilcox Signed-rank Test: W = 49, *p* < 0.001; *N*_*1*_ = *N*_*2*_ = 7). However, this pattern did not emerge for trials with the honey solution treatment in the testing phase (Wilcox Signed-rank Test: W = 12, *p* = 0.13; *N*_*1*_ = *N*_*2*_ = 7).

These data indicated that it is possible to manipulate the value of tuna (reflected by the colony-level foraging decisions) as a contested food resource by supplementary feeding colonies with either tuna or honey solution. The number of workers attending the uncontested food is an index of the value of the food to the colony [54, 55]. Therefore, the value of tuna to a colony is higher if the colony has been previously provided with honey solution, and lower if the colony has been previously provided with tuna. For convenience, the former is referred to as high value and the latter to low value.

### Staged contest experiments with additional compensatory foraging tests

Based on the above results, we decided to use tuna as our treatment food in staged contests, with colony pairs pre-fed either tuna or honey. We selected another 14 colony pairs and subjected them to the pre-feeding phase protocol by splitting them into 2 lots of 7 pairs and pre-feeding with honey solution and tuna pieces separately (see above). Before conducting the staged contest experiment, we performed a compensatory foraging test (see above) to confirm consistent patterns of preferred nutrients. We then conducted, an hour later, staged contest experiments for those selected colony pairs with a recognisable display ground located midway between the nests. For each pair, we placed 30 g of tuna (Woolworths™ branded ‘canned tuna in spring water’, broken into small morsels) in a single place (roughly 5 cm in diameter) on the display ground, ensuring that workers from both colonies would encounter the food at roughly the same time. After 20 min, we placed a raised 30 × 30 cm wooden quadrat above the food and took one digital image of the competing workers located within the boundaries of the quadrat. We used these images to calculate the total number of competing workers within each frame. We also counted, over a one-minute period, the number of workers that were involved in physical ‘grappling’, which often has fatal consequences for either or both contestants [43]. Grappling behaviour rarely occurs during displays but was evident in contests over food. These experiments were repeated using new groups of colony pairs on each separate occasion (February 2020, May 2020, November 2020, February 2021, May 2021).

### Statistical analysis

The individual staged contest experiments that were conducted at different time periods were assigned into one of two season categories: early summer (November, February) and late summer (May). Data for compensatory foraging tests and staged contest experiments were analysed separately. All analyses were conducted using the RStudio Version 1.1.453 platform [56] (Additional files 2, 3).

We used Linear Models to determine whether the patterns of compensatory foraging, reflected by the (log-transformed) number of workers at the feeders, were similar at different times of the year over a two-year period, with pre-feeding treatment, testing treatment, and season (early, late) as fixed effects, and colony as the unit of analysis. Separate models were constructed for each treatment, when there was a significant pre-feeding treatment by testing treatment interaction term, with pre-feeding treatment and season as fixed effects.

The feeding history of the colonies prior to their ‘contest’ may have been slightly compromised by the testing feeding regime: while both neighbouring colonies within each pair had the same pre-feeding regime, they did not necessarily have the same food during the testing regime. Accordingly, we included an additional ‘compromised feeding’ term, with levels of 2 (the most compromised), 1, or 0 (the least compromised) assigned to colony pairs, where different food was used in the pre-feeding and testing phase for both colonies within the pair, in one colony only or in neither colony, respectively. For example, level 0 (no compromise) occurs when both colonies of a pair were fed tuna during both the pre-feeding and testing regime; level 1 (mild compromise) occurs when both colonies were fed tuna during the pre-feeding regime, but one colony was fed tuna and the other colony was fed honey solution during the testing regime; level 2 (most compromised) occurs when both colonies were fed tuna during the pre-feeding regime and fed honey solution during the testing regime. The same rules were applied when the pair of colonies were pre-fed with honey solution.

We used Linear Models to investigate the effects on the (log-transformed) number of workers at the contested food sites, with food value (low, high), compromised feeding (0, 1, 2), and season (early, late) as fixed effects. We used a binomial GLM to investigate the probability of escalation (defined as the observation of any number of grappling individuals), initially with food value (low, high) and season (early, late) as fixed effects. To explore the effect of worker number, we modelled the number of grappling ants (of the total number of ants) as a binomial GLM and used the number of workers in the quadrat as a covariate. Finally, for those contests in which escalated behaviour was observed, we used a Linear Model to investigate the influence of food value and season on the proportion of grappling ants, with worker number as a continuous covariate. We used colony pair as the unit of analysis, and our results reflect an average behaviour of the paired colonies (that received the same food during the pre-feeding regime and the staged contests).

## Results

### Ant behaviour

**Fig. 2:**
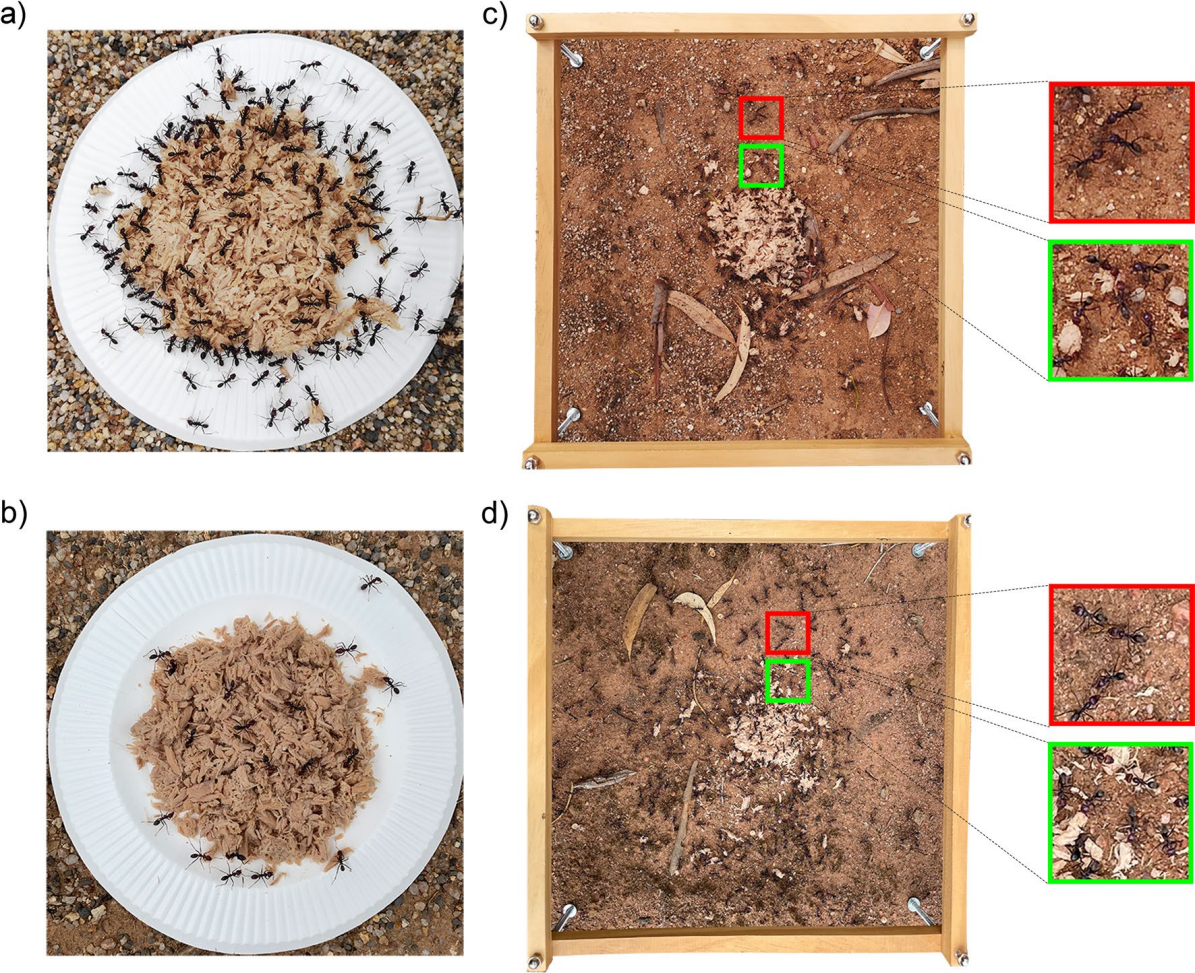
Representative ant behaviours at uncontested food and contested food. **a** and **b** Workers foraging on uncontested tuna placed adjacent to their nests, with a 5-day pre-feeding history of honey **a** or tuna **b**. **c** Workers from paired colonies pre-fed with honey deployed to tuna placed in the display ground. **d** Workers from paired colonies pre-fed with tuna deployed to tuna placed in the display ground

There was a difference in the behaviour of workers at the contested and uncontested food sources (Fig. 2). Most workers attending the uncontested tuna baits on the paper plate, located adjacent to the nest mound, were either foraging on or adjacent to the food or attempting to remove small morsels (Fig. 2a, b). In contrast, the workers attending the contested food very frequently switched tasks between displaying with non-nestmates (see Fig. 2c, d; marked as red) and collecting tuna (see Fig. 2c, d; marked as green), with the displaying area occurring near the tuna.

### Consistent compensatory foraging

**Fig. 3:**
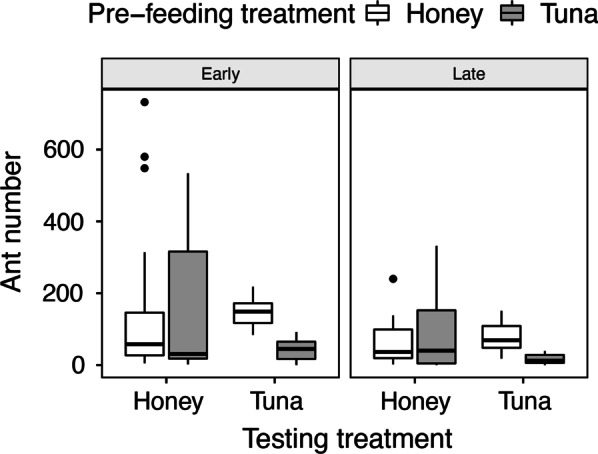
Ant number at uncontested feeders. The number of workers at uncontested feeders with different food (either honey solution or tuna pieces) during the testing phase, following different diets (either honey solution or tuna pieces) during the pre-feeding phase, and at different stages (early and late) of summer (*N* = 140)

The variation in the number of workers of *I. purpureus* at uncontested feeders was explained by a significant pre-feeding phase by testing phase interaction term (F_1,153_ = 12.20, *p* < 0.001; see Additional file 1: Table S1 and Fig. 3), consistent with evidence of compensatory foraging. The pattern was remarkably similar across experiments for the protein (tuna) treatment in the testing phase (see Fig. 3), where the number of ants was significantly higher for colonies previously provided with carbohydrates (honey solution) than with protein (tuna) (t = − 7.93, *p* < 0.001; *N* = 70). In contrast, the number of workers deployed to the honey solution during the testing phase was not influenced by the type of food provided during the pre-feeding phase (t = − 0.65, *p* = 0.52; *N* = 70). Given that the number of ants attending a food source reflects the value of that food source to the workers and colony [54, 55], we deem the value of tuna to the colony to be higher if the colony had been previously fed honey than tuna.

### Effect of food value on contest escalation

**Fig. 4:**
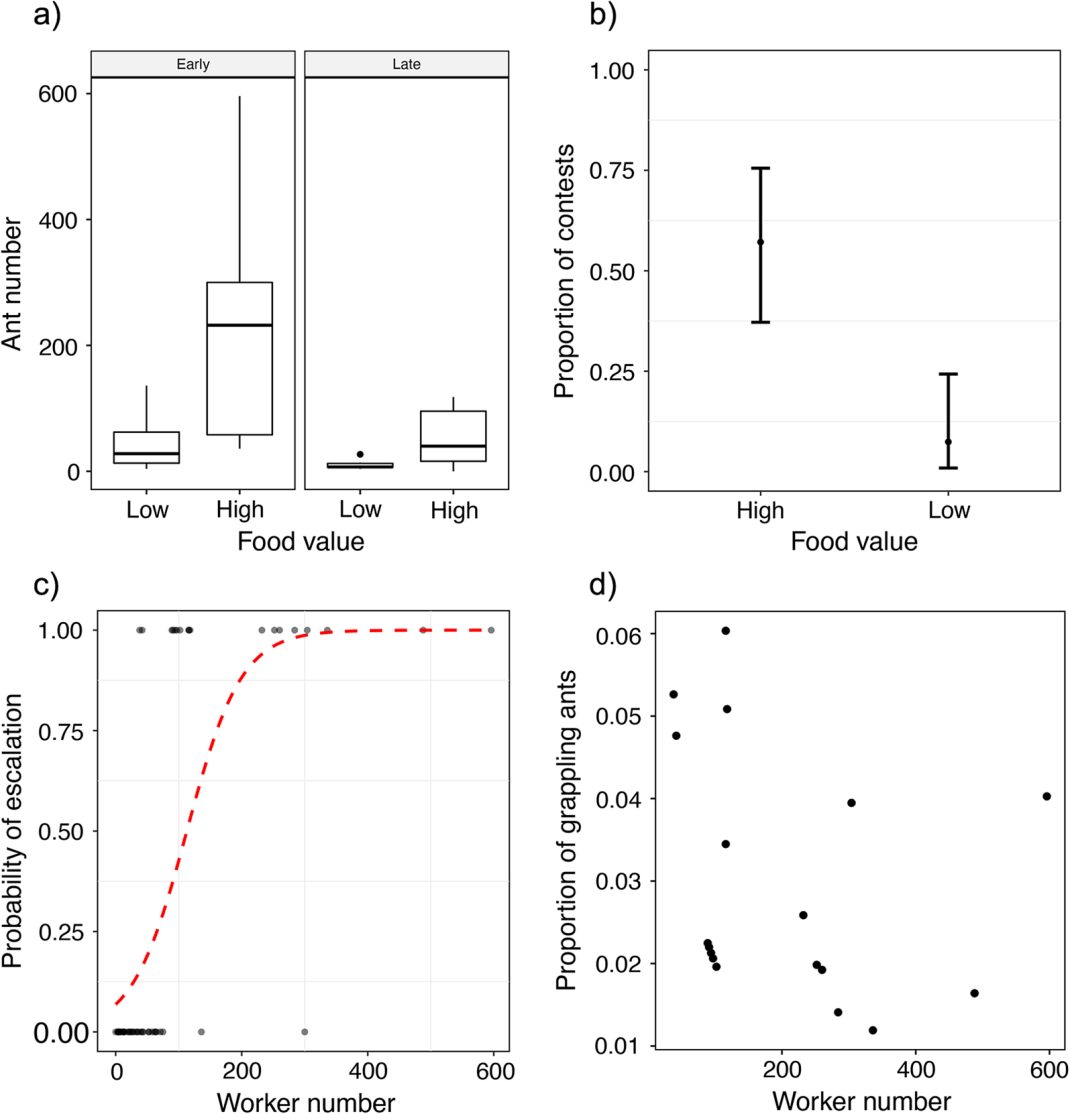
Contest escalation when colony pairs competed for food resources. **a** The number of workers at the display ground contesting food of high (the paired colonies was pre-fed with honey solution) or low (the paired colonies was pre-fed with tuna) value at different stages (early and late) of summer (*N* = 55). **b** The proportion of contests that escalated to grapples when competing over high (the paired colonies was pre-fed with honey solution) or low value food (the paired colonies was pre-fed with tuna) (*N* = 55). **c** The correlation between the probability of escalation and the number of workers at the contesting site (*N* = 55). **d** The proportion of grappling ants is not influenced by the number of workers at the contesting site (*N* = 18). A re-analysis of these data, excluding the point for 596 workers, revealed a weak negative effect of worker number on the rate of (log-transferred) grappling ants (F = 5.4, p = 0.04)

While some display grounds can persist throughout the summer months, others do not. The display ground was not apparent in 15 of the original 70 pairs, and so we conducted staged contest trials with 55 colony pairs. Two lines of evidence indicate that colonies adjust their level of escalation according to the value of the food. First, the number of workers deployed to the contested tuna was greater if the colony had been fed honey solution rather than tuna during the pre-feeding phase (t = − 4.82, *p* < 0.001; *N* = 55; see Additional file 1: Table S2 and Fig. 4a), a pattern that was consistent across the two seasons, but more pronounced during the early season (effect of late season, t = − 4.23, *p* < 0.001; *N* = 55; see Additional file 1: Table S2 and Fig. 4a). We have also tested that these patterns were not influenced by the testing feeding regime (F_2,43_ = 0.0006, *p* > 0.99).

Second, aggressive behaviour among individual workers was more frequently observed when the value of the tuna to the colony was higher: grappling between non-nestmates occurred in 33% of contests (18 out of 55), and the proportion of these escalated interactions in contests over high value food was higher than that over low value food (Z = − 3.15, *p* = 0.002, see Fig. 4b), a pattern that did not differ between seasons (effect of later season, Z = 1.28, *p* = 0.20). This effect was at least partially influenced by the number of workers at the food source: subsequent modelling with worker number as a covariate revealed that the probability of escalation depended on worker number (Z = 2.65, *p* = 0.008, see Fig. 4c). For the subset of contests in which any physical grappling occurred (*N* = 18), the proportion of ants that were grappling did not vary with worker number (F_1,14_ = 1.20, *p* = 0.29, see Fig. 4d), season (F_1,14_ = 0.22, *p* = 0.65) or food value (F_1,14_ = 0.003, *p* = 0.95).

## Discussion

We confirm that a broad principle of animal contest theory, originally developed for dyadic contests, also applies to group contests. Specifically, our field experiments provide compelling evidence that the value of food resources influences the level of escalation in group contests in natural populations of meat ants *I. purpureus*, and that this variation derives from colony-level decisions. Firstly, we successfully developed a protocol for manipulating the value of the contested food resource (morsels of tuna) by altering the dietary histories of the competing colonies. Colonies valued tuna more highly, by deploying more foraging workers [54, 55], if they had been previously provided with a supplementary diet of honey solution (carbohydrates) than tuna (protein), a pattern that was remarkably consistent at different times of the active season across two years. Secondly, in staged contest experiments, neighbouring colonies deployed more workers when contesting high than low value food resources. The ants at the contested food reflect an investment into securing the resource, as any worker deployed to the contested food is very likely to engage with a non-nestmate worker (see Fig. 2c, d) and is thus at risk of injury. Therefore, the number of workers deployed to the contest represents a level of escalation, since it reflects both an opportunity cost (in terms of workers otherwise engaging in other tasks) and a potential workforce loss through mortality. Thirdly, those competitive interactions for higher value food are more likely to escalate to physical grapples, causing severe injuries and even death to both the rivals [43], and this higher level of individual escalation may be triggered, in part, by the frequency of interactions at the competing sites [51].

Significantly, the patterns of escalation reflect the value of the food to the colony, rather than to individual workers. First, the workers competed over tuna, which they do not consume but rather transport to the nest, where it is fed to the larvae [36, 57, 58]. Thus, the value of tuna is likely to be determined by the nutritional requirements of the larval population in the colony. If the contested food was honey, which is consumed by both larvae and workers [35], it would be difficult to distinguish whether the response of workers reflects their physiological circumstances [21] or the nutritional status of the colony. Our study ensures individual workers respond to colony-level signals, which is likely to have originated in larval begging behaviour [59–62], with workers subsequently adjusting worker recruitment using trail pheromones [46, 63, 64] to meet colony-level nutritional needs [42].

Here, the mortality rate was typically low in our group contests, which is not consistent with reports of frequent injuries and deaths in other ant species, including the fire ant *Solenopsis invicta* [65] and the red wood ant *Formica polyctena* [66]. While the frequency with which at least one pair of ants escalated to grappling behaviour was high (57%) when competing over the more highly valued food, the proportion of ants that engaged in this behaviour was low (≤ 0.06). Mortality associated with escalated contests in social insects is thought to have only minimal fitness consequences for the colony as workers represent a dispensable labour force [57], in contrast with group-living vertebrates [67–69]. However, this consistent, low mortality rate may allow colonies of *I. purpureus* to avoid the costs of fighting, when inter-colony disputes over key resources are frequent during active seasons [43, 45, 46, 48].

Many studies have investigated the foraging ecology of ants [55, 70], but there are remarkably few studies of compensatory foraging in natural populations, perhaps because of the challenges of manipulating the macronutrient intake that can be readily enhanced but rarely limited. Our experiments, repeated three times during two summers, reveal a remarkably consistent compensatory response to meet protein targets, while the response to carbohydrate was more varied in both degree and direction (see Additional file 1: Table S3 and Fig. S1). These differences likely reflect a consistent, limited availability of protein in the natural habitat of *I. purpureus*, which contrasts with the relatively ready availability of carbohydrates that are accessed through honeydew secreting hemipterans [46–48, 50]. Nutrient preference assays across a range of species of ants with different diets reveals a general preference for nutrients that are less accessible [40, 41], while a review of field and laboratory studies suggests a preference for the more commonly consumed nutrient [55]. The pattern of compensatory foraging response in field populations of the congener *I. suchieri* [40] suggest a stronger response to protein than carbohydrate targets, although the pattern was less pronounced than that of *I. purpureus*. In contrast, laboratory studies of *I. mayri* [71] reveal a response to carbohydrate but not protein targets. The variable response to carbohydrate targets across our replicated field experiments cautions against drawing strong inferences from experiments that are not replicated, since the compensatory response is likely to vary with both temporal changes in colony growth and environmental conditions.

## Conclusions

The Geometric Nutritional Framework provides a useful tool for designing experiments that test whether the value of food influences contest escalation. Specifically, it avoids introducing other factors (individual motivation, metabolism rate, lactic acid level) that may otherwise enhance [21, 72–74] or constrain [75] individual fighting behaviour after a period of food deprivation. This experimental method for manipulating resource value could be used in many other field-based empirical studies of contest theory involving both dyadic and group contests. More importantly, our experimental design ensured that the contest escalation reflects collective decisions. First, individual physiological effects were removed because the contested food was not consumed by the workers, who transported it to the larvae, so the observed variation in individual fighting behaviour precisely reflects colony-level nutritional demand. Second, we utilised field experiments to investigate group contests in social insects, allowing individual workers to be connected with their own nests and thus respond to colony signals.

## Supplementary Information

**Additional file 1**: **Table S1**. The Linear model investigating the sources of variation in the number of workers at uncontested feeders with different food during the testing phase, following different diets during the pre-feeding phase, and at different stages of summer. **Table S2**. The Linear model investigating the sources of variation in the number of workers at competing sites over high or low value food, and at different stages of summer. **Table S3**. The Linear model investigating the sources of variation in the number of workers at uncontested feeders with honey solution during the testing phase, following different diets during the pre-feeding phase, and at all different times across two years. **Fig. S1**. Ant number and foraging history across two years. The number of workers attending the feeder during the testing phase, following different nutritional histories during the pre-feeding phase, and at all different times across two years.

**Additional file 2.** R script associated with statistical analysis.

**Additional file 3.** Dataset associated with statistical analysis. Sheet 1. The numer of ants at uncontested feeders with different food during the testing phase in 2019 Nov. Sheet 2. The number of ants with different prefeeding history at uncontested feeders with different food in 2019 Nov. Sheet 3. The number of workers at uncontested feeders with different food at different stages of summer. Sheet 4. The number of workers at uncontested feeders with tuna pieces during the testing phase. Sheet 5. The number of workers at uncontested feeders with honey solution during the testing phase. Sheet 6. The number of workers at competing sites over high or low value food at different stages of summer. Sheet 7. The number of grappling ants and grappling rate in escalated contests.

## Data Availability

The data and relevant code are provided in the Additional files associated with this manuscript.
