# Supplementary material for: Colony-level aggression escalates with the value of food resources
Source: BMC Ecol Evol. 2023 May 16;23:18. doi: 10.1186/s12862-023-02117-x (PMC10189932; doi:10.1186/s12862-023-02117-x)
Supplement: Supplementary file 1 — Additional file 1: Table S1. The Linear model investigating the sources of variation in the number of workers at uncontested feeders with different food during the testing phase, following different diets during the pre-feeding phase, and at different stages of summer. Table S2. The Linear model investigating the sources of variation in the number of workers at competing sites over high or low value food, and at different stages of summer. Table S3. The Linear model investigating the sources of variation in the number of workers at uncontested feeders with honey solution during the testing phase, following different diets during the pre-feeding phase, and at all different times across two years. Fig. S1. Ant number and foraging history across two years. The number of workers attending the feeder during the testing phase, following different nutritional histories during the pre-feeding phase, and at all different times across two years. [file 12862_2023_2117_MOESM1_ESM.docx]

**Additional Information**

**Table S1.** The Linear model investigating the sources of variation in the number of workers at uncontested feeders with different food (either honey solution or tuna pieces) during the testing phase, following different diets (either honey solution or tuna pieces) during the pre-feeding phase, and at different stages of summer (*N* = 150).

|  | Estimate | | SE | | df | Statistics | | Probability | |  |
| --- | --- | --- | --- | --- | --- | --- | --- | --- | --- | --- |
| *Parameter estimates* |  | |  | |  | t Ratio | | >\|t\| | |  |
| Terms |  | |  | |  |  | |  | |  |
| Intercept | 4.29 | | 0.23 | |  | 18.51 | | < 0.001 | |  |
| Season(late) | -0.79 | | 0.22 | |  | -3.61 | | < 0.001 | |  |
| Pre-feeding (tuna) | -0.24 | | 0.30 | |  | -0.80 | | 0.43 | |  |
| Testing feeding (tuna) | 0.69 | | 0.30 | |  | 2.29 | | 0.02 | |  |
| Pre-feeding (tuna): Testing feeding (tuna) | -1.50 | | 0.43 | |  | -3.49 | | < 0.001 | |  |
| *Fixed effects tests* | |  | |  | |  | F | | >F | |
| Main effects | |  | |  | |  |  | |  | |
| Season |  | |  | | 1 | 13.02 | | < 0.001 | |  |
| Pre-feeding |  | |  | | 1 | 21.33 | | < 0.001 | |  |
| Testing feeding |  | |  | | 1 | 0.07 | | 0.80 | |  |
| Pre-feeding: Testing feeding |  | |  | | 1 | 12.20 | | < 0.001 | |  |

**Table S2.** The Linear model investigating the sources of variation in the number of workers at competing sites over high or low value food, and at different stages of summer (*N* = 55).

|  | Estimate | | SE | | df | Statistics | | Probability | |  |
| --- | --- | --- | --- | --- | --- | --- | --- | --- | --- | --- |
| *Parameter estimates* |  | |  | |  | t Ratio | | >\|t\| | |  |
| Terms |  | |  | |  |  | |  | |  |
| Intercept | 4.87 | | 0.24 | |  | 19.97 | | < 0.001 | |  |
| Season(late) | -1.36 | | 0.32 | |  | -4.23 | | < 0.001 | |  |
| Food value (low) | -1.45 | | 0.30 | |  | -4.82 | | < 0.001 | |  |
| *Fixed effects tests* | |  | |  | |  | F | | >F | |
| Main effects | |  | |  | |  |  | |  | |
| Season |  | |  | | 1 | 12.83 | | < 0.001 | |  |
| Food value |  | |  | | 1 | 23.24 | | < 0.001 | |  |

**Table S3.** The Linear model investigating the sources of variation in the number of workers at uncontested feeders with honey solution during the testing phase, following different diets (either honey solution or tuna pieces) during the pre-feeding phase, and at all different times across two years (*N* = 84).

|  | df | Statistics | Probability |
| --- | --- | --- | --- |
| *Fixed effects tests* |  | *F* | *>F* |
| Main effects |  |  |  |
| Year | 1 | 26.65 | < 0.001 |
| Time | 2 | 6.21 | 0.003 |
| Pre-feeding treatment | 1 | 0.01 | 0.91 |
| Year: Time | 2 | 6.85 | 0.002 |


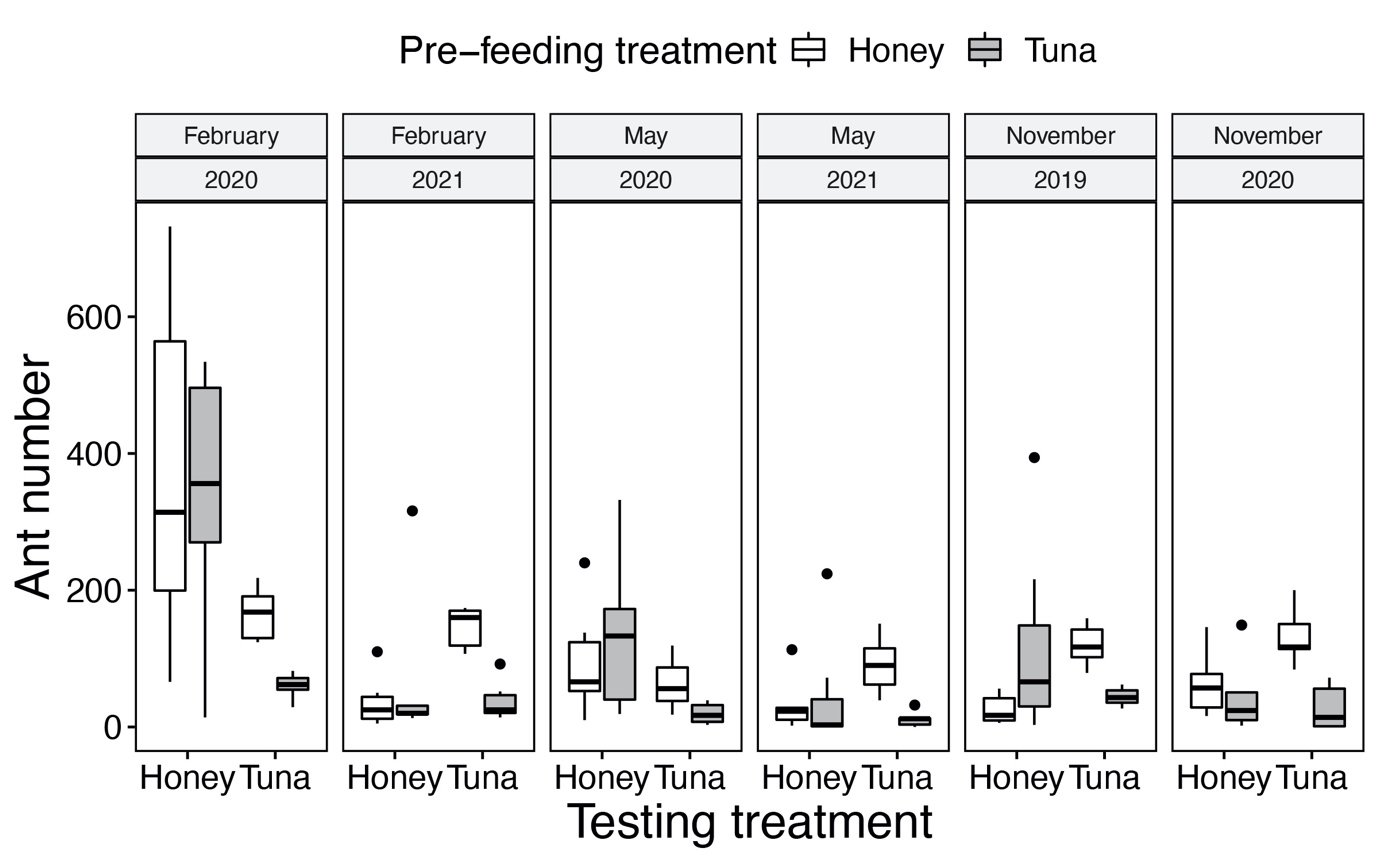


**Fig. S1.** Ant number and foraging history across two years. The number of workers attending the feeder (either honey solution or tuna pieces) during the testing phase, following different nutritional histories (either honey solution or tuna pieces) during the pre-feeding phase, and at all different times across two years (*N* = 168).
